# Urethral caruncle in a 9-year-old girl: a case report and review of the literature

**DOI:** 10.1186/s13256-015-0518-7

**Published:** 2015-03-28

**Authors:** Masahiro Chiba, Akira Toki, Akihide Sugiyama, Rie Suganuma, Shunsuke Osawa, Rie Ishii, Tomokazu Nakagami, Junichi Suzuki, Yu Watarai, Shinya Kawano, Koumei Suzuki

**Affiliations:** 1 Division of Pediatric Surgery, Department of Surgery, Showa University School of Medicine, Showa University Hospital, 1-5-8 Hatanodai, Shinagawa-ku, Tokyo 142-8666 Japan; 2 Children’s Medical Center, Showa University Northern Yokohama Hospital, 35-1 Chigasaki-chuo, Tsuzuki-ku, Yokohama-shi, Kanagawa-ken 224-8503 Japan; 3 Children’s Medical Center, Showa University Koto Toyosu Hospital, 5-1-38 Toyosu, Kouto-ku, Tokyo 135-8577 Japan

**Keywords:** Caruncles, Child, Female, Urethral

## Abstract

**Introduction:**

Urethral caruncles are the most frequent benign tumors of the female urethra. Most of them are found in post-menopausal women, and they are rare in childhood. Only a few pediatric cases have been published in the literature. In this report, we present an unusual case of a pediatric patient with a urethral caruncle, along with a review of the literature.

**Case presentation:**

A 9-year-old Mongolian girl was referred to our hospital with a 2-week history of frequent adherence of a small amount of blood to her underwear. We found a sessile smooth margin, a clear boundary and an elastic, soft red tumor over the entire circumference of the urethral meatus. At the beginning, because of the child’s age, urethral prolapse was suspected. There was no response after 3 weeks of conservative treatment with steroid ointment. With the patient under general anesthesia, a partial tumor resection was performed for the purpose of histological examination. The tumor excision was limited to about 1/2 laps of the urethral meatus to prevent the development of urethral stricture. On the basis of clinical and histopathological examinations, a diagnosis of a urethral caruncle was made. Post-operatively, steroid ointment application to residual masses was continued, and these disappeared about 6 months later. Our patient was free of recurrence and had had no complications after 3 years of follow-up.

**Conclusions:**

Urethral caruncles are rare in children, and the possibility of malignancy is slight during this period. Biopsy of the mass is not required for diagnosis. It should be indicated only if the mass has other characteristics that raise suspicion of malignancy. In previously reported cases, all of the tumor was removed. However, the trigger of the caruncle in childhood is chronic inflammation. Conservative therapy with steroid ointment should be the core treatment. However, it may be necessary to proceed to treatment because caruncles take a long time to heal. The case that we describe in this report will serve as an example for similar cases in the future.

## Introduction

Urethral caruncles are benign and pedunculated and appear as sessile polypoid lesions of the posterior lip of the urethral meatus. They represent the most common lesion of the female urethra and occur primarily in post-menopausal women [1,2]. Only 14 pediatric cases have been published in the English-language literature to date [3-6]. In this report, we present a case of a urethral caruncle in a 9-year-old girl and review the relevant literature.

## Case presentation

**Figure 1:**
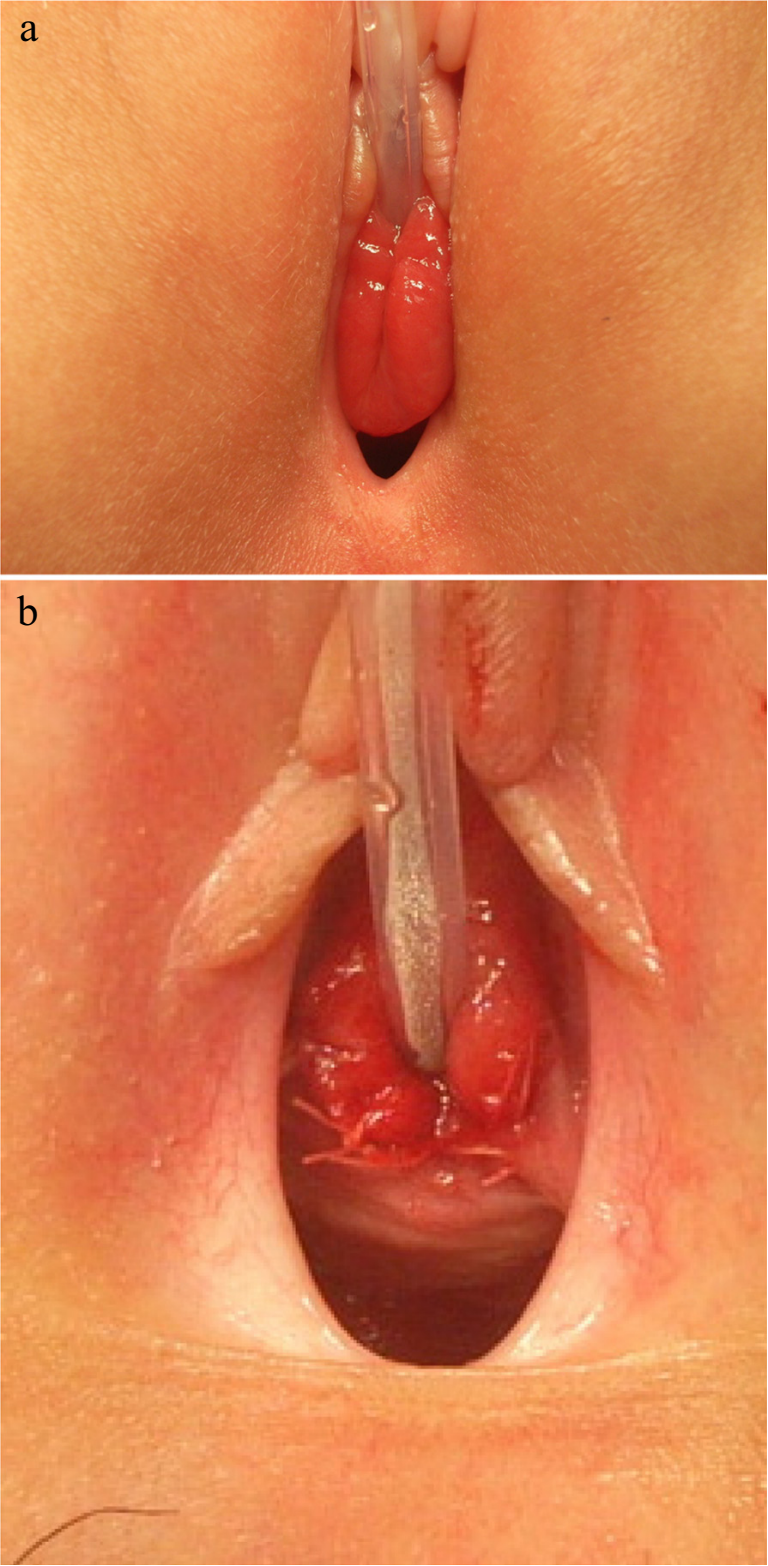
**Photographs showing the pre- and post-operative appearance of the lesion. (a)** Pre-operative photograph showing an elastic, soft red tumor originating from the urethral meatus. **(b)** Post-operative appearance of the surgical site. The dorsal mass excision was about 1/2 laps of the urethral meatus. (tumor resection: 10×15mm).

**Figure 2:**
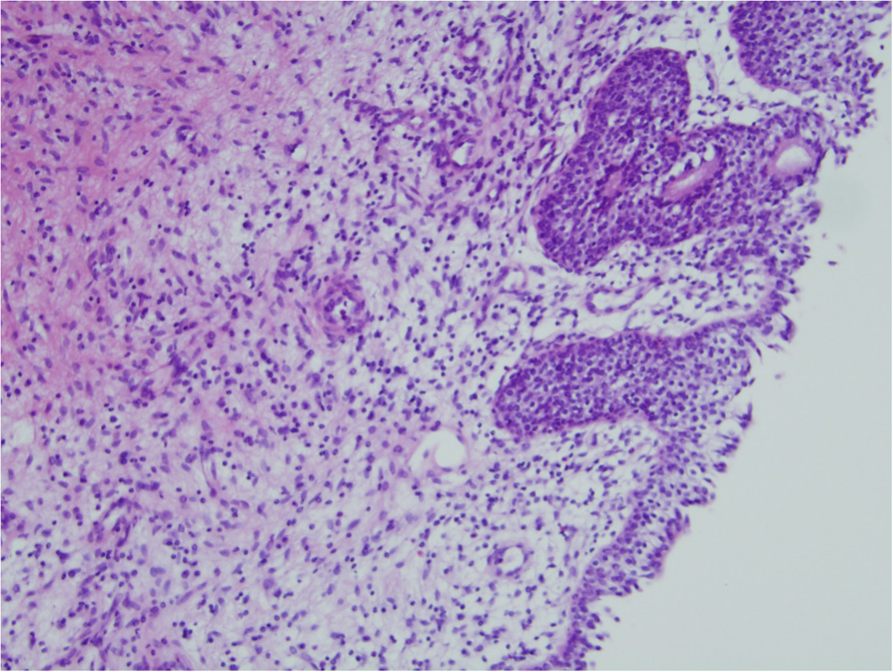
**Microscopic image of the patient’s tumor.** Histological stain shows interstitial edema with inflammatory cell infiltration, papillary hyperplasia of the connective tissue and coverage with stratified squamous epithelium (hematoxylin and eosin stain; original magnification, ×100).

A 9-year-old Mongolian girl was referred to our hospital with a 2-week history of frequent adherence of a small amount of blood to her underwear. The girl’s mother’s pregnancy and the girl’s birth and neonatal history were uneventful. She had no history of hospitalization or surgery. We found a sessile smooth margin, a clear boundary and an elastic, soft red tumor over the entire circumference of the urethral meatus (Figure 1a). The patient experienced no pain or difficulty with urinating, although the tumor was hemorrhagic. No other abnormalities were found during the physical examination. All of the results of the complete blood count and biochemical examinations were normal. At the beginning, because of the girl’s age, urethral prolapse was suspected. No clinical response was achieved after 3 weeks of conservative treatment with steroid ointment. The exclusion of malignant tumors such as rhabdomyosarcoma and malignant lymphoma was also required, so we performed enhanced computed tomography. We observed no muscle layer invasion or distant metastases. With the patient under general anesthesia, a partial tumor resection was performed for the purpose of histological examination. The tumor excision was limited to about 1/2 laps of the urethral meatus, 10×15mm in size, to prevent the development of urethral stricture (Figure 1b). A histological examination revealed interstitial edema with inflammatory cell infiltration, papillary hyperplasia of the connective tissue and coverage with stratified squamous epithelium. There was no evidence of malignant change throughout the specimen (Figure 2). On the basis of the clinical and histopathological examinations, the diagnosis of a urethral caruncle was made. Post-operatively, steroid ointment application to residual masses was continued, and these disappeared about 6 months later. Our patient was free of recurrence and had no complications after 3 years of follow-up.

## Discussion

Urethral caruncles are usually confined to the female urethra. They most often occur in middle-aged and elderly women and are rarely found in young females [1,2]. A total of 14 cases of young females have been described in the English-language literature [3-6]. Because the details of the 11 cases that Campbell reported [3] are unknown, only 4 pediatric cases, including ours, are summarized in Table 1. The polypoid lesions in pediatric cases were present at the urethral meatus in all patients; in adult patients, they occur most frequently at the posterior lip. Unlike adult cases, bleeding was the only symptom other than persistent pain or difficulty with urinating in this case. In adult patients, chronic inflammation can also be present, in addition to atrophy and curing of the urethral mucosa due to decreased secretion of estrogen [7]. Therefore, a variety of symptoms associated with reduced secretion of estrogen appear in adults, but they differ in children. The trigger of the caruncle in childhood is also chronic inflammation; however, the exact etiology is unknown. The female urethra is particularly susceptible to infection as well as to inflammation. Further study of the pathogenesis of urethral caruncles may be required.

**Table 1:** **Summary of four cases reported in the literature**

**Patient**	**Sex/age**	**Site/size**	**Treatment**	**Histological type**	**Prognosis**
1	F/2 yr, 5mo	Posterior lip of mid-urethra/0.6cm	1 wk conservative management, complete resection	Vasodilatation type	No recurrence for 2 yr
2	F/9 yr	Anterior lip of urethra/1.0cm	Complete resection	Vasodilatation type	No recurrence for 2 yr
3	F/2 yr, 7mo	Posterior lip of urethra/0.7cm	Complete resection	Granulomatous type (included intestinal heterotopia)	Unknown
Our patient	F/9 yr	All around the circumference of urethra/2.0cm	3 wk conservative management, partial resection, 6 mo conservative management	Granulomatous type	No recurrence for 3 yr

F; female. yr; year. wk; week. mo; month.

The clinical differential diagnosis for a urethral or peri-urethral mass in pediatrics is urethral prolapse and peri-urethral gland abscess. Although uncommon, a spectrum of neoplasms may mimic urethral caruncle clinically, including sarcoma [8]. Biopsy of the mass is not required for diagnosis, but it should be indicated if the mass is irregular or has other characteristics that raise suspicion of malignancy.

The management of such patients can be divided into conservative treatment and surgical resection. Conservative therapy with application of estrogen ointment is common in adults [9], and steroid ointment is used in children because chronic inflammation is also a trigger. In previously reported cases, short-term steroid ointment treatment was ineffective and all of the tumor was ultimately removed. Recurrences have also been seen in adult cases [4], but the curability of these tumors with surgical resection is high in pediatric patients. No cases of recurrence have been reported (Table 1). However, if the lesion covers the entire circumference of the urethral meatus, as in our patient, meatal stenosis after surgical excision is a potential problem [10]. Therefore, in our patient, we restricted the surgery to a partial resection and made a pathological diagnosis after the procedure. After surgery, we used a long-term treatment period of 6 months, and the patient’s urethral caruncle was completely cured with steroid ointment. In conservative treatment, it may be necessary to proceed to treatment, considering the fact that these lesions take a long time to heal.

## Conclusions

Urethral caruncles are rare in children, and the possibility of malignancy is slight during this period. Biopsy of the mass is not required for diagnosis. Biopsy should be indicated only if the mass has other characteristics that raise suspicion of malignancy. In previously reported cases, all of the tumor was removed. However, the trigger of caruncles in childhood is chronic inflammation. Conservative therapy with steroid ointment should be the core treatment. However, it may be necessary to proceed to treatment because these lesions take a long time to heal. Because this disease is rare in children, the treatments employed for our patient and in the other cases reported in the literature should be borne in mind when such patients are encountered. The case that we describe in this report will serve as an example for similar cases in the future.

## Consent

Written informed consent was obtained from the patient’s legal guardian for publication of this case report and any accompanying images. A copy of the written consent is available for review by the Editor-in-Chief of this journal.
